# Home-Based Rehabilitation With Frenkel’s Exercises for Adrenomyeloneuropathy: A Case Report

**DOI:** 10.7759/cureus.80878

**Published:** 2025-03-20

**Authors:** Takemasa Ishikawa

**Affiliations:** 1 Nana-r Home-Visit Nursing Development Center, Tekix Corporation, Osaka, JPN

**Keywords:** adrenomyeloneuropathy (amn), frenkel’s exercises, home-based care, home-visit nurse, rehabilitation, x-linked adrenoleukodystrophy (x-ald)

## Abstract

Adrenoleukodystrophy (ALD) encompasses a spectrum of X-linked genetic disorders, including the adult-onset adrenomyeloneuropathy (AMN) phenotype. We report the case of a 32-year-old male with AMN and examine the impact of a tailored home-based rehabilitation regimen, specifically employing Frenkel’s exercises. The patient displayed classic AMN symptoms, including spastic paraparesis, peripheral neuropathy, and urinary disturbances. Initial assessments revealed significant ataxia-related impairments, particularly in gait, stance, and heel-shin slide. A home-visit nurse collaborated with the patient to develop and implement a personalized regimen utilizing Frenkel’s exercises. Over one month, the patient demonstrated remarkable improvement, reducing his Scale for the Assessment and Rating of Ataxia score from 10 to 5. Detailed analysis revealed specific enhancements in gait, stance, and fine motor control, aligning with the goals of Frenkel’s exercises. These findings suggest that Frenkel’s exercises can be a valuable addition to AMN rehabilitation, addressing ataxia-related motor deficits. While recognizing AMN’s complexity and individual variability, further research is essential to refine rehabilitation strategies. Long-term follow-up studies are needed to assess intervention sustainability and its impact on the ALD spectrum’s overall disease progression and quality of life.

## Introduction

Adrenoleukodystrophy (ALD) encompasses a spectrum of X-linked genetic disorders stemming from mutations in the *ABCD1* gene, which lead to the accumulation of very long-chain fatty acids [1]. This accumulation results in a variety of phenotypes, one of which is adrenomyeloneuropathy (AMN), typically presenting in adulthood with a progressive course of spastic paraparesis, peripheral neuropathy, and often adrenal insufficiency. AMN is considered a milder adult-onset phenotype of ALD, but it, nonetheless, poses significant challenges to those affected, often leading to a gradual decline in mobility and daily functioning [2]. In Japan, as of 2023, there were 249 registered AMN patients, with an estimated prevalence of 1 in 20,000 to 30,000 males [3].

The rehabilitation needs of patients with AMN are complex and ongoing due to the progressive nature of the disease [4]. While rehabilitation is considered important in managing AMN, there is a paucity of reports on the specific effectiveness of various rehabilitation approaches. Physical therapy is a cornerstone of AMN management, aiming to maintain mobility and prevent secondary complications associated with spasticity and immobility [2]. Given the progressive loss of motor and sensory functions, rehabilitation in AMN is not only necessary but must be adaptable to the changing needs of the patient over time.

Frenkel’s exercises have demonstrated potential as a rehabilitation approach for addressing ataxic movements observed in patients with balance disorders, including AMN, originating from either vestibular or peripheral neuropathy causes [5,6]. These exercises, originally conceptualized by Heinrich Frenkel [7], are specifically designed to engage the central nervous system through meticulous repetition and are performed in three distinct postures, namely, seated, lying down, and standing. Key elements of these exercises include concentration, repetition, and precision, aiming to improve balance and reduce the risk of falls by stimulating the visual, somatosensory, and vestibular systems.

In the context of this progressive and debilitating condition, the role of the home-visit nurse becomes crucial. Home-visit nurses are uniquely positioned to provide consistent, tailored care that addresses the specific needs of patients with AMN. They can monitor the patient’s condition, adjust care plans as needed, and deliver interventions such as physical therapy exercises that are essential for maintaining function and quality of life. In Japan, rehabilitation is primarily provided by physiotherapists and other rehabilitation professionals. However, in certain home-care settings where physiotherapy services are limited, nurses may also be involved in implementing structured exercise programs, including Frenkel’s exercises.

This case report aims to shed light on the potential benefits of a home-based rehabilitation program led by a home-visit nurse for a patient with AMN. It details the implementation of Frenkel’s exercises, a therapeutic modality traditionally used for ataxia, and discusses its efficacy in improving AMN coordination and proprioceptive deficits. By presenting this case, we hope to contribute valuable insights into the rehabilitation of AMN within the ALD spectrum.

## Case presentation

A 32-year-old male, presenting with symptoms indicative of AMN, the adult-onset phenotype of ALD, was evaluated for progressive motor and sensory impairments. Genetic testing confirmed a pathogenic mutation in the *ABCD1* gene, supporting the diagnosis of AMN. The patient provided informed verbal consent to include the case in this report. He lived independently and reported a history of motor difficulties since childhood, including a subjective perception of slow gait, meaning difficulty keeping pace with others in daily activities, along with challenges in fine motor tasks such as drawing circles. He had become increasingly prone to tripping and experienced progressive difficulty with walking from the age of 27, necessitating the use of walls and a cane for support.

The patient also reported non-motor symptoms, including impotence and urinary disturbances, which had developed alongside his motor symptoms. Cognitive assessment with the Mini-Mental State Examination yielded a full score of 30/30, indicating no global cognitive impairment. The Frontal Assessment Battery resulted in a score of 17/18, with a single deduction for verbal fluency.

Neurological examination revealed diminished Achilles tendon reflexes and reduced reflexes in the biceps, triceps, and knee jerk, which were suggestive of peripheral neuropathy [8]. Sensory examination demonstrated decreased tactile sensation below the right L5 dermatome and loss of vibratory sensation at the right medial malleolus and hallux. However, joint position sense remained intact in the thumbs of both the upper and lower limbs. Notably, spasticity was present in the right lower limb, but manual muscle testing indicated normal strength (grade 5) in all limbs. MRI examination showed mild cerebellar atrophy, with no other significant abnormalities observed. Considering these findings, the patient’s gait disturbance was attributed to a combination of cerebellar ataxia, as evidenced by mild cerebellar atrophy on MRI, and peripheral neuropathy, which manifested as sensory deficits in the lower limbs. The patient had no known family history of AMN or other related neurological conditions.

The Scale for the Assessment and Rating of Ataxia (SARA, Japanese version) was employed to quantify the severity of the patient’s ataxia [9]. SARA is a clinical tool specifically designed to measure the degree of ataxia, which is a lack of voluntary coordination of muscle movements. The scale consists of eight items that evaluate functions such as gait, stance, sitting, speech, finger chase test, nose-finger test, fast alternating hand movements, and heel-shin test. Each item is scored individually, and the sum provides an overall score ranging from 0 (no ataxia) to 40 (most severe ataxia). SARA is widely used in clinical settings and research to assess the severity and progression of ataxia in various neurological disorders [10,11].

**Figure 1:**
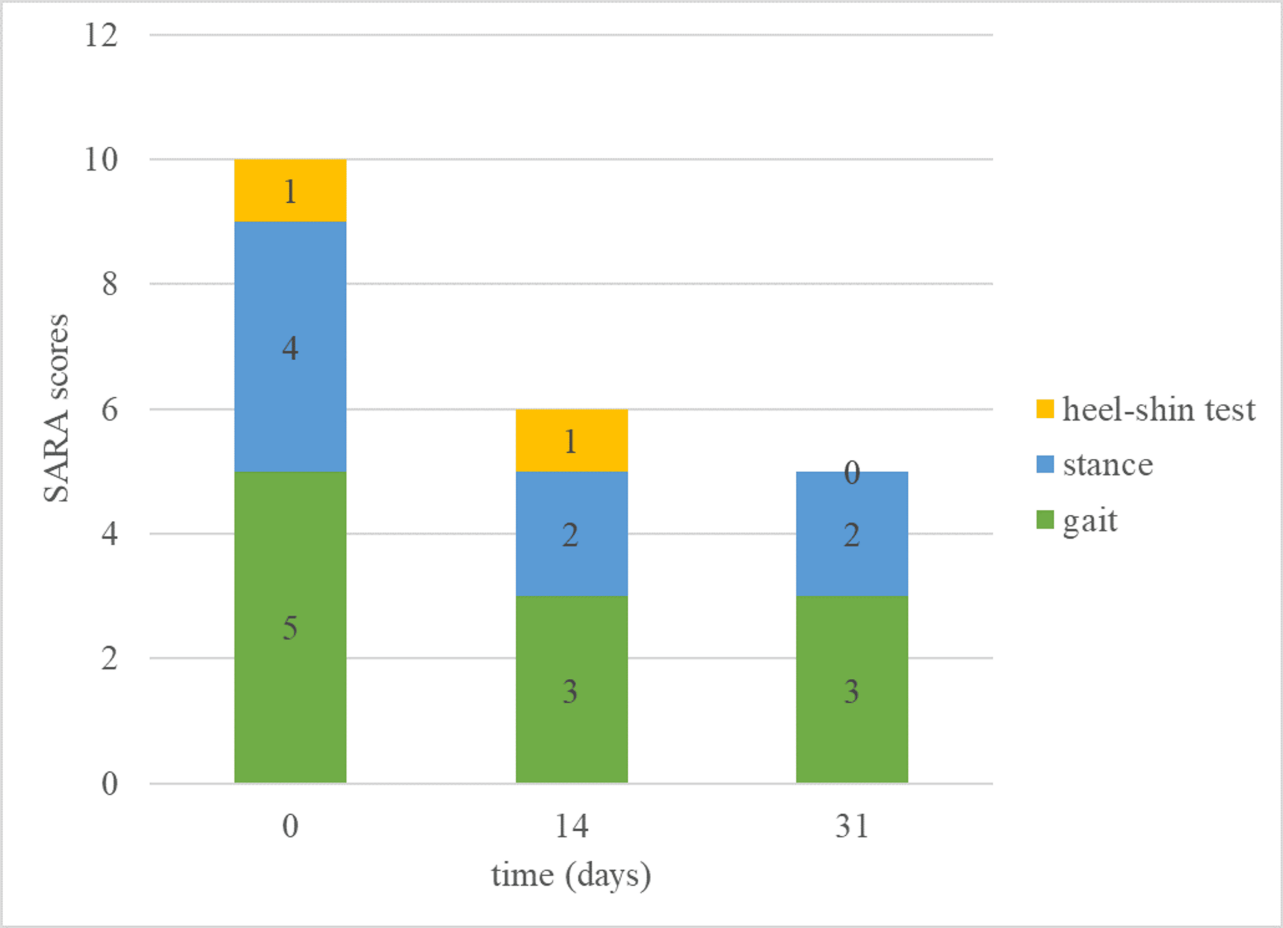
The SARA score changes over a month in an AMN case. SARA: Scale for the Assessment and Rating of Ataxia; AMN: adrenomyeloneuropathy

Figure 1 shows the change in the patient’s SARA score between the initial assessment and one month later. Initial SARA evaluation yielded a score of 10, with significant impairments in gait (5 points), stance (4 points), and heel-shin slide (1 point).

Following the initial SARA evaluation, which yielded a score of 10, the home-visit nurse developed a personalized self-training regimen to target the patient’s ataxic movement disorders specifically. This regimen incorporated Frenkel’s exercises, chosen for their efficacy in improving coordination and proprioception, which are often affected in ataxic patients. To illustrate the specific exercises implemented in this case, we refer to the Physiopedia resource on Frenkel’s exercises, which provides detailed descriptions and visual representations of these movements [12]. The patient actively participated in developing the regimen to tailor it to his specific needs and make it practical for daily practice. After one month of dedicated self-practice with these exercises, his SARA score improved to 5, demonstrating notable improvements in gait and stance, and a normalization of the heel-shin slide test.

Video 1 demonstrates the walking status one month after rehabilitation. Before the rehabilitation, the patient could only walk with support along the wall, but as shown in the video, he has now attained an improved walking ability.

**Video 1:** The walking condition following one month of rehabilitation. This video demonstrates the patient’s gait before and after rehabilitation.

## Discussion

In the case of ALD, AMN presents a distinct clinical challenge due to its progressive nature, encompassing a spectrum of motor and sensory impairments [4]. The patient in this case report exhibited classic symptoms of AMN, including spastic paraparesis and peripheral neuropathy. These findings are in line with the broader clinical picture of ALD, where neurological decline can significantly impact the quality of life [13].

The significant improvement in his SARA score from 10 to 5 highlights the effectiveness of Frenkel’s exercises in enhancing motor function. Similar improvements have been reported in studies investigating Frenkel’s exercises, particularly in patients with cerebellar ataxia and sensory ataxia due to peripheral neuropathy [14,15]. These studies demonstrate that Frenkel’s exercises can enhance coordination and proprioception, leading to functional improvements. To gain a deeper understanding of the impact of Frenkel’s exercises, we delved into a detailed analysis of the specific SARA components where improvements were observed.

Gait (initial score: 5 points, final score: 3 points): The gait component was one of the most significant improvements in the patient’s SARA score. This outcome suggests that Frenkel’s exercises positively affected his walking abilities. The patient’s initial difficulty with walking and reported tendency to trip had likely stemmed from impaired coordination and proprioception, common features of ataxic disorders. The exercises, designed to promote coordination and relearn motor skills, seem to have been particularly effective in improving his gait.

Stance (initial score: 4 points, final score: 2 points): Another substantial improvement was observed in the stance component of SARA. This result indicates that the exercise regimen also benefited the patient’s ability to maintain balance and stability while standing. The exercises likely enhanced his proprioceptive feedback, enabling better control over his posture.

Heel-shin slide (initial score: 1 point, final score: 0 points): The normalization of the heel-shin slide test further underscores the effectiveness of Frenkel’s exercises. This particular test assesses the patient’s ability to smoothly move their heel along the shin of the opposite leg, a task requiring precise coordination and proprioception [16]. The patient’s progression from a score of 1 to 0 in this category reflects a significant improvement in his fine motor control and proprioceptive capabilities.

These observed improvements in gait, stance, and fine motor control are consistent with the objectives of Frenkel’s exercises, which aim to enhance coordination and proprioception, the deficits commonly encountered in patients with ataxic disorders such as AMN [17,18]. Frenkel’s exercises have been reported to improve balance disorders caused by ataxic movements [15,17]. In this case, the improvement in balance was observed to lead to enhanced scores in the gait and stand scores. Furthermore, Frenkel’s exercises have been documented to be effective in alleviating cerebellar ataxia [14]. In this case, it is posited that the improvement in cerebellar ataxia contributed to the enhanced scores observed in the heel-shin test. The exercises encourage slow, repetitive movements that promote learning motor skills through neuroplasticity. In the case of our patient, this approach appears to have effectively addressed the coordination and proprioceptive deficits associated with AMN, resulting in tangible improvements in his daily motor function. However, existing literature suggests that Frenkel’s exercises may not be the most effective intervention for all types of balance impairments. For instance, vestibular rehabilitation and dynamic balance training have shown superior outcomes in specific populations [19]. While these alternatives may provide greater improvements in some cases, Frenkel’s exercises offer the following distinct advantages: they are simple, require no specialized equipment, and can be easily incorporated into a home-based rehabilitation program. In this case, the patient was able to integrate these exercises into his daily routine with minimal assistance, which likely contributed to the observed improvements in gait, stance, and fine motor control.

These findings suggest that Frenkel’s exercises can be a valuable addition to the rehabilitation toolkit for patients with AMN, particularly in addressing specific motor deficits related to ataxia and coordination disorders. Frenkel’s exercises are designed to improve coordination and proprioception through repetition-based movement training, which is particularly relevant for AMN patients presenting with both cerebellar ataxia and peripheral neuropathy. It is important to recognize that AMN is a complex and progressive condition and individual responses to these exercises may vary.

## Conclusions

This case with AMN highlights the potential of home-based rehabilitation, particularly through structured coordination exercises such as Frenkel’s exercises. These exercises provide a simple and accessible approach to improving motor function, requiring no specialized equipment and allowing for self-directed practice. The observed improvements in coordination and balance suggest that targeted rehabilitation can play a significant role in maintaining functional mobility and enhancing the quality of life for individuals with AMN. Home-visit nurses are well-positioned to support such rehabilitation efforts, especially in settings where access to physiotherapy services may be limited. While this case suggests the feasibility of Frenkel’s exercises as part of AMN rehabilitation, further research is necessary to evaluate their long-term effectiveness and compare them with other rehabilitative strategies. Establishing evidence-based guidelines for individualized rehabilitation programs will be essential in optimizing outcomes for patients with AMN and other neurodegenerative conditions.
